# The role of mitochondrial RNA association for mitochondrial homeostasis in neurons

**DOI:** 10.1042/BCJ20230110

**Published:** 2024-01-29

**Authors:** Inmaculada Segura, Angelika Harbauer

**Affiliations:** 1 Max Planck for Biological Intelligence, Neurometabolism Group, 82152 Planegg-Martinsried, Germany; 2 Biomedical Center Munich, Department of Cellular Physiology, Ludwig-Maximillian-University, 82152 Planegg-Martinsried, Germany; 3 Institute of Neuronal Cell Biology, TUM School of Medicine, Technical University of Munich, 80802 Munich, Germany; 4 Munich Cluster for Systems Neurology, 81377 Munich, Germany

**Keywords:** cotranslational import, hitch-hiking, import, mitochondria, mRNA localization, mRNA transport, ribosome, translation

## Abstract

The sub-compartmentalization of cellular processes is especially important in highly polarized cells such as neurons, as their function rely on their complex morphology. The association of RNAs to the mitochondrial surface is a conserved feature from yeast to humans and it regulates several aspects of mitochondrial physiology and, hence, cellular functions. In neurons, mitochondria are emerging as platforms for RNA transport and local protein translation. In this review, we discuss how RNA localization to mitochondria helps to sustain mitochondrial function, and how this can support mitochondrial homeostasis, especially in the distal parts of the neuron, to support neuronal activity.

## Introduction

Creating order from chaos is an energy-demanding task, no matter whether we talk about a toddler's room or the crowded environment within the cellular cytoplasm. With the origin of eukaryotic cells 2 billion years ago, compartmentalization of cellular tasks was possible by sorting of specific proteins into membrane-enclosed organelles. To achieve this challenging task, multiple protein import machineries have evolved. These protein complexes recognize specific signals on their intended protein cargos. Mitochondria, the endosymbiotic ‘power plant of the cell’, are no exception despite containing their own genome. This small remnant of their bacterial origin only encodes for 13 proteins in humans, barely 1% of their estimated proteome [1]. Thus, proper targeting of mitochondrial protein precursors to this essential organelle is necessary for their proper function. Mitochondria are not only the main suppliers of cellular ATP, but they play a role in many other metabolic reactions as well as cellular signaling events, ranging from lipid metabolism and Ca^2+^ buffering to apoptosis and innate immune signaling [2]. Neuronal physiology is particularly dependent on mitochondria, as they almost exclusively use oxidative phosphorylation (OXPHOS) to generate ATP [3]. This high demand may be connected to their high energetic demand due to constant membrane re-polarization, Ca^2+^ transients and synaptic transmission. Mitochondrial defects, therefore, are often associated with neurological phenotypes, and mitochondrial dysfunction is a hallmark of neurodegenerative diseases, including Parkinson's disease (PD) [2]. Hence, proper targeting of mitochondrial protein precursors to successfully replenish neuronal mitochondria is paramount to preserve neuronal health.

In neurons as in all other eukaryotic cell types, the import of mitochondrial protein precursors is achieved by a set of import pore complexes. They are located in the mitochondrial outer membrane (OMM) and inner membrane (IM), termed translocase of the outer/inner membrane (TOM/TIM), respectively [4]. The receptors of the TOM complex Tom20/22 and Tom70 recognize either alpha-helical, amphipathic N-terminal signal sequences or internal, mostly hydrophobic mitochondrial targeting signals (MTSs), respectively [5–7]. The later proteins are shielded from cytoplasmic aggregation by association with molecular chaperones such as the heat shock protein 70 (Hsp70), which binds to the TOM receptor to hand over its cargo [6,7]. Decades of elegant *in vitro* studies have shown that, unlike import into the endoplasmic reticulum (ER) lumen, mitochondrial protein import can occur in a post-translational manner [8,9]. This is contrasted with early findings that detected ribosomes attached to the mitochondrial surface [10], which suggested that even though mitochondrial protein import *in vitro* can occur post-translationally, the cell might also use localized translation and even co-translational import to help correct protein sorting *in vivo* [11]. The idea of localized translation at the mitochondrial surface was further supported by the identification of selective enrichment of transcripts encoding mitochondrial proteins in purified mitochondria [12]. More recently, proximity biotinylation methods either specifically targeting mitochondrial localized ribosomes and their associated transcripts [13,14] or mitochondrial localized RNAs [15–17] further expanded the set of transcripts located near the mitochondrial surface *in vivo*. Interestingly, these more targeted approaches also contributed to identify some transcripts encoding non-mitochondrial localized proteins that were also located to mitochondria, suggesting that transcripts destined to other cellular locations may use mitochondria as platforms for RNA transport and/or translation. *Vice versa*, there is also evidence for transcripts encoding mitochondrial-destined proteins that are translated at cytosolic or even at ER-associated ribosomes [16,18,19], arguing that there must be specific mechanisms that target some, but not all mitochondrial mRNAs to their destination organelle.

## Modes of mRNA association to mitochondria

In general, most studies have identified two modes of mitochondrial association of mRNAs: (i) Targeting of transcripts during active translation, by a mechanism that can be blocked with translation inhibitors that destabilize the translating ribosomes and (ii) ribosome-independent tethering due to their interaction with mitochondria-associated RNA-binding proteins (RBPs). Understanding which proteins directly tether the mRNA or the translating ribosome along with its RNA template and the emerging nascent polypeptide to mitochondria is an active area of study that so far has revealed different mechanisms used by either unicellular yeast or higher eukaryotes. However, while the tethering proteins are not conserved between yeast and humans, some general principles seem to apply.

### RBP-mediated association

**Figure 1.**
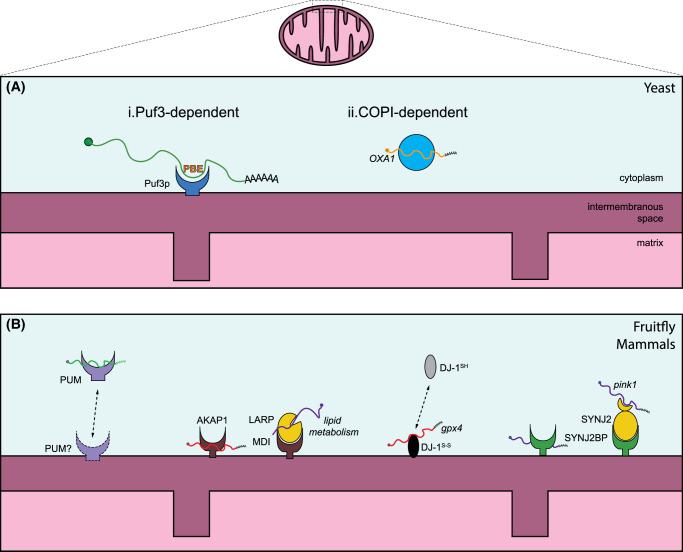
Mechanisms of mRNA recruitment to mitochondria, independent of the ribosome. (**a**) Mechanisms identified in yeast to recruit mRNAs encoding mitochondrial proteins to the outer mitochondrial membrane include (i) the Puf3p protein that recognizes Puf-binding elements (PBEs) sequences in target transcripts and (ii) the COPI transport of mRNAs. (**b**) Mechanisms identified in fruitflies and mammalian cells include (i) Pumilio/PUM mediated recruitment, (ii) AKAP1-mediated recruitment, either directly or via its binding partner LARP, (iii) the recruitment by DJ1, that is regulated by its redox status and (iv) SYNJ2BP-mediated recruitment either directly or via its protein binding partner SYNJ2.

Visual evidence for mitochondrial association of RNAs comes from *in situ* hybridization studies or studies that tag the transcript of interest with the MS2 aptamer, a phage-derived repetitive RNA element, that is specifically recognized by the capside MS2-coat protein (MCP) which is expressed tagged with a fluorescent protein [18,19]. For instance, the MS2/MCP system has been used in budding yeast to analyze the subcellular distribution of 24 different transcripts encoding proteins covering all mitochondrial sub-compartments [20]. Consistent with a specificity in mitochondrial targeting, many (but not all) transcripts displayed a moderate to high degree of colocalization with mitochondria. In yeast, the RBP Puf3p was described to mediate subcellular localization of RNAs [21,22], and both overexpression and deletion of Puf3p affect both yeast growth on non-fermentable media and mitochondrial respiratory activity [21,23]. Accordingly, the deletion of Puf3p partially prevents the mitochondrial association of mRNAs containing a ‘Puf3p-binding element’ (PBE; consensus sequence UGUANAUA) [20] (Figure 1a). However, it is unclear how much of those functional effects are mediated by altering the mitochondrial association of mRNAs *per se*, as Puf3p has also been shown to negatively affect the polyadenylation and stability of its targets [24] as well as to be important for proper motility and morphology of mitochondria in yeast [23]. The partial effect of Puf3p deletion on mRNA localization suggests that there might be redundant systems that could also associate mRNAs to mitochondria. One of these alternative mechanisms that directly recruits mRNAs to mitochondria in yeast is the coat protein complex (COPI) [25] (Figure 1a). The COPI complex is part of the intracellular vesicular transport system, and it is necessary for the localization of certain nuclear mRNAs coding for mitochondrial proteins to the mitochondrial surface [25]. Also in murine motor neuron-like NSC34 cells, COPI-mediated transport of certain mRNAs has been described [26]. Among them, several mitochondrial mRNAs were identified [26]. These mRNAs were enriched in G-quadruplex motifs and binding partners of the RBP Fragile X mental retardation protein (FMRP), potentially connecting the COPI-mediated maintenance of mitochondrial RNA association to the function of this RBP mutated the *FMR1* gene (coding FMRP) in Fragile X mental retardation [27] and hence to neuronal health.

In metazoans, the Puf3p homologs Pumilio2 (PUM2 in mammals) and PUF-8 (in *Caenorhabditis* *elegans*) are up-regulated during aging and they negatively regulate the translation of the mitochondrial fusion protein Mitochondria fission factor (MFF), leading to increased mitochondrial fission in older mice and worms [28]. Moreover, *cross*-*linking* and immunoprecipitation analysis revealed that PUM2 binds to numerous mRNAs that are key for mitochondrial functions (both in the brain and in the spleen), to repress their expression [29]. This is antagonized by the long noncoding RNA *Norad*, which regulates the derepression of those transcripts [29]. While this shows that the molecular function of Puf3p is most likely conserved, it is unclear whether this includes the targeting of homologous transcripts to mitochondria, as mammalian PUM2 is rather located in cytoplasmic ribonucleoprotein (RNP) granules [30,31] (Figure 1b), that in some cases colocalized with processing bodies (P-bodies) markers [32]. Physiological PUM2-containing RNP granules in the colon cancer cell line HCT116 cells are formed by liquid–liquid phase separation and contain *Norad* [30], but metabolic stress in neurons relocates PUM2 to stress granules [33]. Other conserved cytoplasmic RNP granules that repress nuclear-encoded mitochondrial mRNAs include Smaug bodies. Experiments in U2OS cells have shown that Smaug bodies can sporadically contact with the surface of mitochondria [34]. The dissolution of Smaug bodies is regulated by mitochondrial activity, and enables the release of its complexed mRNAs to be translated, hence regulating mitochondria respiration and morphology [34]. In neurons, synaptic activity also induces the dissolution of Smaug bodies, regulating synaptic plasticity [35], although it is not clear whether this is mediated by mitochondrial functions.

Further RBPs involved in the association of mRNAs to the mitochondrial surface have been identified in metazoans, such as DJ-1, AKAP1, SYNJ2BP and SYNJ2a [17,36–38]. Among them, the A-kinase anchoring protein 1 (AKAP1, also known as AKAP149/121 or dAKAP1) is better characterized. As other AKAPs, AKAP1 can bind to protein kinase A (PKA), but it is unique as it is located at the mitochondrial outer membrane and contains an RNA-binding motif [39,40]. Due to its different domains, AKAP1 can regulate mitochondria in different ways. For instance, upon PKA recruitment, AKAP1 can induce effects on mitochondrial morphology and function (reviewed in [41]). As an RBP, AKAP1 preferentially binds to transcripts involved in lipid and steroid metabolism (Figure 1b), which explains why the role of AKAP1 as an RBP has mainly been studied in adipocytes or adrenocortical carcinomal cell lines. Its best characterized RNA-binding partner is the transcript encoding the steroidogenic acute regulatory (StAR) protein [42], but it can also bind to the transcripts encoding the manganese-dependent superoxide dismutase, some subunits of the respiratory chain and tricarboxylic acid cycle (TCA) enzymes, or the transcript encoding the ER-targeted lipoprotein lipase (LPL) [40,42–44]. In mammalian cells, it was shown that PKA-mediated phosphorylation of AKAP1 (e.g. downstream of catecholamine signaling or by the introduction of a phospho-mimetic mutation) induces the tethering of the *ATPase subunit Fo-f* mRNA to mitochondria [40] and increased translation of *LPL* mRNA [43], presumably on mitochondria-associated ribosomes. AKAP1 also recruits the translational activator La-related protein 4 (LARP4) to mitochondria [37]. LARP4 is phosphorylated by AKAP1-associated PKA as well [37], which may explain the increase in translation of AKAP1-bound mRNAs upon PKA stimulation. The interaction between AKAP1 and LARP as well as the translational regulation of mitochondrial-associated mRNAs are conserved in *Drosophila melanogaster* [45] (Figure 1b)*.* In *Drosophila* ovaries, local translation of Larp-associated transcripts at the mitochondrial surface is important for the amplification of mitochondrial DNA (mtDNA). Loss of MDI, the fly ortholog of AKAP1, leads to mtDNA-deficient mature eggs [45]. Interestingly, targeting Larp to the mitochondrial outer membrane independently of MDI was enough to rescue the mtDNA defect [45], suggesting that the mRNA-binding activity of AKAP1/MDI may be dispensable in this system. In neurons, however, it remains to be determined whether AKAP1 is mediating mitochondrial RNA association. Interestingly, overexpression of AKAP1 has been already studied in the context of neurodegenerative diseases as a potential neuroprotective mechanism (reviewed in [41]), due to its positive effect on mitochondrial health and neuronal survival. Among others, the loss of PTEN-induced kinase 1 (PINK1) function, a hereditary cause of recessive familiar PD, can be rescued by overexpression of AKAP1 [46]. Much of this neuroprotective function may derive from the increase in local PKA activity and its inhibitory effect on mitochondrial fission [47]. Interestingly, a role for the RNA-binding activity of AKAP1 to regulate mitochondrial morphology has also been described [48]. As the mRNA-encoding PINK1 has recently also been shown to be located to the mitochondrial surface ([38], see below), it is tempting to speculate that also *Pink1* mRNA association and/or translation at mitochondria may be beneficially influenced by AKAP1 overexpression. Most likely, AKAP1 is a multifunctional scaffolding protein, whose various functions all synergistically contribute to maintain mitochondrial morphology and function in neurons.

A further multifunctional protein with a reported RNA-binding capacity is the PD-associated protein DJ-1 [36]. DJ-1 is mainly present in the cytosol, but can be recruited to the mitochondria upon oxidation [49,50] (Figure 1b). The mutation of the oxidized cysteine residue (Cys 106) in DJ-1 prevents its neuroprotective function [51]. Transcripts bound to DJ-1 encode proteins targeted to mitochondria, including glutathione peroxidase 4 (GPX4) [36]. GPX4 is a key protein in glutathione (GSH) synthesis, an antioxidant protein that prevents the iron-dependent, oxidative cell death pathway called ferroptosis [52]. Overexpression of DJ-1 inhibits ferroptosis by stimulating the GSH production for the transsulfuration pathway [53]. It has been suggested that upon oxidative stress, DJ-1 dissociates from its RNA cargos, allowing their translation, e.g. during ischemia and reperfusion stress [54]. Higher GPX4 synthesis together with the increased production of other proteins encoded by DJ-1-associated transcripts may provide further protection against cell death pathways. Ferroptosis may specifically be relevant in dopaminergic neurons of the *substantia nigra* [55], tying the function of DJ-1 in regulating ferroptosis to the most vulnerable cell type in PD.

Finally, proximity biotinylation assays in human cells in culture not only confirmed the presence of nuclear mRNAs coding for mitochondrial proteins associated with the outer mitochondrial membrane, but also identified or confirmed RBPs located at that membrane [16,17]. One of these RBPs is Synaptojanin 2 binding protein (SYNJ2BP), a PDZ-binding protein that is anchored to the outer mitochondrial membrane via its C-terminal helix. A previous screening in human cells in culture identified SYNJ2BP as an RBP [56], and further analysis revealed that SYNJ2BP can recruit specific mRNAs to the mitochondrial surface [17] (Figure 1b). Interestingly, some mRNA-binding partners of SYNJ2BP remained localized at the mitochondrial surface upon inhibition of translation or heat-induced stress, suggesting that SYNJ2BP would protect them and keep them at the proper subcellular localization for local translation after release of the inhibition [17]. Interestingly, similar to the stacking of RBPs with MDI and AKAP1, SYNJ2BP interacts with another RBP, a specific splice variant of Synaptojanin 2 (SYNJ2, [38,56]) (Figure 1b). Unlike SYNJ2BP, which binds mRNAs in both translation-dependent and translation-independent ways, SYNJ2 has so far only been implicated in stabilizing the association of mRNAs to mitochondria targeted in a translation-dependent manner and will, therefore, be discussed in the next chapter.

### Translation-dependent association

**Figure 2.**
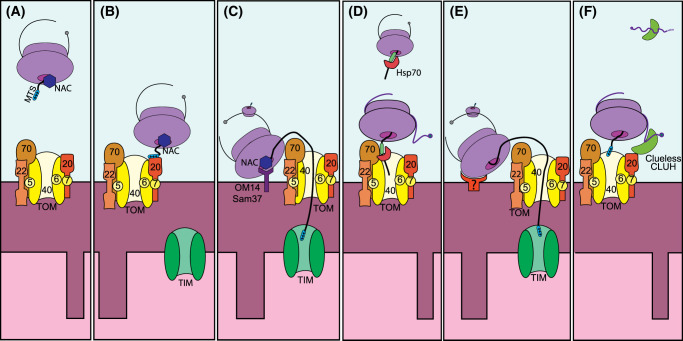
Mechanisms of mRNA recruitment to mitochondria, dependent on ribosomal translation. (**a**) Cytoplasmic ribosomes translating mitochondrial proteins are recruited to the outer mitochondrial membrane by different mechanisms. (**b**) The N-terminal MTS is engaged to Tom40, the pore protein of the TOM complex, allowing co-translational import. Proteins targeting the inner membrane or the matrix will be imported by the translocase of the inner membrane (TIM) complex. (**c**) For the stabilization and/or recruitment of the translating complexes, the receptors OM14 or Sam37 interact with the NAC complex. (**d**) Internal hydrophobic sequences in the synthesizing polypeptide are recognized by the chaperone heat shock protein 70 (Hsp70) and recruited to the TOM70 receptor. This recruitment is either co- or post-translational. (**e**) Direct stabilization of the translating ribosome. (**f**) Recruitment of specific mRNAs to the OMM by CLUH/Clueless mediated interaction.

Although in some contexts, the transport of mRNAs to their subcellular compartment is independent of their translation, it also has been reported that the association of the translating ribosome with the target organelle leads to the localization of the mRNA close to the subcellular destination of the newly synthesized protein [11,57]. This process is best known at the ER, but translation near other membranous organelles (such as mitochondria) has also been observed [58,59]. In the case of the mitochondria, more than half of its nuclear-encoded mitochondrial proteins carry N-terminal mitochondrial targeting sequences, which would allow the interaction of the nascent chain with the TOM complex during translation (Figure 2a,b). This interaction could then be stabilized by interactions between mitochondrially localized RBPs, ribosomal receptors or receptors for the chaperones associated with either the nascent chain or internal hydrophobic sequences [11,60,61] (Figure 2c–f). Examples will be explained in more detail in the following chapters. A further, less explored alternative is that heterogeneity within the composition of the ribosome itself, such as the inclusion of alternative ribosomal protein paralogues. The replacement of individual ribosomal subunits by their paralogues has already been shown to impact cellular respiration in yeast [62]. This can also lead to the formation of ribosomes with preferential subcellular locations. This is seen in heart tissue, in which the ribosomal protein of the large subunit 3 (RPL3) is replaced by a longer isoform (RPL3L), that reduces the ribosomal interactions with mitochondrial proteins [63]. The ratio of the two paralogues can be altered in disease, such as during heart hypertrophy or infarct, and might serve as a translational switch to regulate mitochondrial function under stress [63]. Further work is needed to understand how RPL3L drives this association on a molecular level and whether ribosomal heterogeneity based on different paralog composition is a general principle regulating mitochondrial RNA association in other cell types.

In yeast, ribosomal association with mitochondria is limited. A few ribosomes have been visualized by electron cryo-tomography on isolated mitochondria [64], but their number was greatly enhanced upon translation arrest with cycloheximide, an inhibitor that blocks translation progression but stabilizes the interaction between ribosomes and their mRNA substrate. Stalled ribosomes were located in discrete clusters near the crista junctions in cryo-tomograms, both as monosomes and polysomes, and their exit tunnels were directionally oriented toward the OMM [64], supporting a co-translational mitochondrial import. The interaction between ribosomes and yeast mitochondria is insensitive to GTP-induced instability when translating ribosomes are synthesizing an MTS-containing polypeptide [61], suggesting that the import of the MTS would anchor and stabilize the translating complex. Interestingly, the development of improved segmentation methods based on machine learning systems allowed the identification of ribosomes associated with mitochondrial surface, in an orientation that is compatible with the association of the nascent polypeptide-associated complex (NAC) [65]. This co-translational import has been shown to be very efficient, especially for proteins of the inner mitochondrial membrane [11,13]. It is proposed that kinetic constraints should limit the association of translating ribosomes with mitochondria, as co-translational import would otherwise slow down fast-dividing cells, impairing their doubling of mitochondrial proteome on time before every cell division [66]. Yet given the slower division rate of mammalian cells, and especially the post-mitotic status of neurons, co-translational import into mitochondria may be a more frequent event in metazoans than in fungi.

A special case is the biogenesis of proteins that are localized in two different subcellular locations, one of them being the mitochondria. In the case of yeast fumarase, a TCA cycle enzyme that is located in both the mitochondria and the cytosol, it has been proposed that it is first locally translated and co-translationally imported into mitochondria. Here, mitochondrial peptidases will cleave its MTS and then, processed fumarase would be retrotranslocated to the cytosol [67]. Recent evidence has shown that mitochondrial recruitment and import relies on its MTS, not only to mediate co-translational import, but also to be properly expressed in the cytosol [68]. Exclusive localization of fumarase of yeast mitochondria by adding the gene to the mtDNA revealed a crucial function for its cytosolic version in the DNA damage response [69], highlighting the importance of the dual localization of this enzyme. It remains to be determined if the distribution of fumarase or other dually localized proteins can be influenced by altering the association of their mRNAs to mitochondria. Also, because the mammalian fumarase probably uses two alternate translation products instead of this co-translational mechanism reported in yeast [70], it needs to be determined whether maybe other, dually localized proteins take advantage of co-translational import for their proper subcellular distribution.

#### Chaperone-mediated ribosomal association with mitochondria

The nascent polypeptides that emerge from translating ribosomes are recognized by the cytoplasmic NAC, a chaperone that regulates their subcellular distribution. In yeast, the NAC recognizes the nascent MTS and regulates mitochondrial protein destination [71]. In the absence of active NAC (alone or combined with Mft52p/MFT1 loss), yeast shows aberrant mitochondria *in vivo* due to defective protein targeting to mitochondria, suggesting that NAC would protect the nascent peptides from aggregation or degradation and enhance proper targeting [71]. In eukaryotes, the NAC complex is heterodimeric. Functional NAC complexes are essential, as knockout animals for the genes encoding its units are lethal [72–74]. In yeast, however, different combinations of heterodimers have been described and single deletion of their subunits are not lethal [71]. Different NAC heterodimers not only show non-identical substrate binding and different subcellular distribution, they also bind to alternative receptors in the mitochondrial outer membrane. The outer membrane protein 14 kDa (OM14) is the receptor for ab-NAC complexes, while sorting and assembly machinery 37 (SAM37) recruits ab’-NAC complexes [75,76]. Although the structure of NAC and their receptor complex with the ribosome at the mitochondria has not been formally shown, evidences in tomographies obtained from yeast cells suggest that they would anchor the ribosomes to the mitochondrial OMM in a tilted position [65]. Moreover, mitochondrial complexome analysis confirmed the presence of OM14 at the OMM either associated with the TOM complex or the outer mitochondrial protein 45 (OM45) [77]. Both OM14 and SAM37 would facilitate the recruitment of translationally active ribosomes to the mitochondria. In the case of the ab’-NAC complexes, the target nascent chains contain a cluster of positively charged amino acids within an amphipathic helix in their N-terminus, such as in Oxa1 or Fum1 [68]. In humans, the homolog protein for OM14 has not been identified, raising the question which molecular receptor would be involved. The role of Metaxin 1, however, the mammalian homolog for SAM37, has been mainly studied in the context of protein import, but whether it also associates with the NAC complex and recruits translating ribosomes is yet to be determined [78].

#### Tom-dependent ribosomal association with mitochondria

The TOM complex is composed by several subunits, where Tom40 builds the pore across the OMM to import proteins into the mitochondria. On the other hand, Tom20, Tom22 and Tom70 act as receptors for different mitochondrial-targeted proteins that will be transferred to the pore Tom40, while the small subunits Tom5, Tom6 and Tom7 stabilize the complex. Although the TOM subunits do not interact directly with mRNAs, they are involved in the mitochondrial localization of certain transcripts. The two receptors differ in their substrate specificity and mode of binding to the preproteins: While Tom20 directly interacts with the MTS of mitochondrial protein precursors, Tom70 first interacts with the chaperones bound to mitochondrial protein precursors carrying internal targeting sequences, mostly of the mitochondrial carrier protein family [4]. Genetic deletion of the receptors Tom20 or Tom70, among others, reduces the mitochondrial localization of mitochondrial mRNAs in yeast [20]. In human cells in culture, however, TOM70 silencing reduces the recruitment of ribosomes translating its targets, but increases the recruitment of TOM20 targeted proteins [14], suggesting that either TOM20 competes with TOM70 for TOM40 or that TOM20 increase is due to a compensatory mechanism. Nevertheless, the silencing of TOM20 induced lethality in similar experiments [14]. TOM70-mediated recruitment depends on protein chaperones that preserve the proteins ready for the import. The protein chaperon Ssa1 in yeast, at least in part, also regulates the mitochondrial localization of mitochondrial mRNAs [79]. Such recruitment is, most probably, due to direct interaction with the hydrophobic region in the translating peptide (and not via mRNA–Ssa1 interaction), targeting the translating complex to the receptor Tom70 [79]. Interestingly, deletion of Ssa1 affects the distribution of mRNAs, not only to the mitochondria, but also to the ER [79], suggesting that this type of cytosolic chaperones can sort the destination of translating ribosomes according to signaling peptides encoded in the nascent polypeptide, and not only based on signals included in the transcripts. In mammalian cells, the chaperones Hsp70 and Hsp90 deliver to Tom70 the mitochondrial proteins containing internal MTSs, but this is mainly done in a post-translational manner, instead of recruiting mRNAs/ribosomes to the mitochondria [59].

Individual features such as coding length, translation speed, MTS maturation and diffusion speed of the precursor from the ribosome to the import complexes will determine the degree of mRNA localization at mitochondria in yeast [80]. It was shown experimentally that translation pausing can also regulate mRNA recruitment and co-translational import on the nascent chain, by a mechanism called Mito-ENCay [81]. Yeast growing in fermentative conditions activate Mito-ENCay as a quality control mechanism to avoid the overexpression of, at least, *MMF1* mRNA, coding for a mitochondrial matrix protein. Here, the Ccr–Not4 complex pauses the ribosomes translating *MMF1* and dock them at the mitochondrial outer membrane via the NAC–Om14/Sam37–MTS–Tom20 docking complexes, and inducing *MMF1* mRNA degradation [81]. How mammalian cells and especially neurons use the properties of translational regulation and MTS maturation to regulate the amount of co-translational import will be an intriguing field of further studies.

On the other side, as soon as nascent peptides that contain an N-terminal MTS interact with Tom20, this may lead to co-translational import and hence RNA association with mitochondria. In mammalian cultured cells, proximity biotin labeling experiments revealed that ribosomes translating mitochondrial proteins were located in the proximity of Tom20 [14], supporting this co-translational import. Deletion of Tom20 in yeast reduces the repertoire of mRNAs associated with mitochondria, although the degree of the effect is transcript dependent [82]. The recruitment of these mRNAs is further impaired in yeast double defective for Tom20 and Puf3, suggesting that both mRNA sequences (via Puf3p interaction) and protein sequences (the MTS via Tom20) co-operate for the proper recruitment of mitochondrial proteins, mitochondrial function and cell survival [82]. In *Drosophila*, it has been suggested that this interaction between Tom20 and the nascent chain is supported by direct interactions of the RBP Clueless with both Tom20 and the ribosome [83]. Clueless/CLUH is a conserved RBP found in yeast (Clu1), plants (Friendly, FMT), flies (Clueless) and mammals (CLUH), and in all organisms it is required for the proper cellular distribution of mitochondria [84–87]. While a mitochondrial localization has been shown for Clueless in flies and it has been suggested for CLUH in mammals, Clueless/CLUH also form phase-separated cytosolic granules in all analyzed species [84–86]. These granules have been termed ‘bliss particles’, as they are only present in conditions of nutrient abundance and in the absence of oxidative stress [85], suggesting that their function might be connected to the metabolic well-being of the cell.

Fitting to the role of CLUH in the regulation of mitochondrial transcripts, depletion of CLUH in mammalian cells alters metabolism [31,88,89]. While some reports in hepatocytes and mouse embryonic fibroblasts argue that CLUH stabilizes mRNA upon binding [89], a recent study failed to observe this effect in both mouse embryonic stem cells and the colon cancer cell line HCT116 [90], suggesting that this function of CLUH might depend on its cellular context. Interestingly, the latter study used proximity biotinylation for CLUH combined with proteomics and could identify in the cytosol transient interactions between CLUH and mitochondrial preproteins prior to their import, in a translation inhibitor-sensitive manner [90]. This favors the model that also mammalian CLUH is involved in the targeting of mitochondrially destined precursor proteins to mitochondria and its association with their mRNAs may only occur in the context of the translating ribosome (Figure 2f). This is supported by ribosomal proximity labeling surrounding mitochondria in HeLa cells, in which silencing of CLUH reduced the overall amount of ribosomes near mitochondria [14]. Interestingly, several mRNAs of potential CLUH-targets remained associated with mitochondria and even increased their abundance in mitochondrial isolations, most likely in an untranslated state [14]. Therefore, the absence of CLUH does not affect the overall translation efficiency of its target mRNAs, but decreases their translation in the vicinity of mitochondria [90]. How these CLUH-target mRNAs are targeted to mitochondria in the absence of either translation or CLUH-binding remains to be determined. CLUH is broadly expressed, and its knockout is embryonically lethal in mice [89], highlighting the importance of this mitochondria-associated RBP for survival. However, CLUH expression in the brain is relatively low [89] and its role in mitochondrial homeostasis in neurons still needs to be established.

#### Co-targeting of transcripts through translation and RBP-mediated RNA binding

As mentioned above, some cooperation between translation-dependent mRNA targeting to mitochondria and the recognition of the mRNA by an RBP is already evident by the synthetic lethality of Puf3p and Tom20 in yeast [82]. This dual targeting mechanism is most likely the case when the anchoring of an mRNA to the mitochondrial surface required two different regions of the sequence, one encoding the MTS and the other containing the RBP binding site. This is the case for the yeast *ATP2* mRNA, which encodes for the beta subunit of the F1-ATP synthase. This transcript relies on sequences both included within the 3′-UTR and the coding sequence to be anchored to the outer mitochondrial membrane [91,92]. The substitution of these sequences by equivalent ones of other mRNAs produced defective ATP synthase and respiratory deficient strains, suggesting the requirement of specific RBPs and/or co-translational import processes. In subcellular fractions obtained from rat hepatocytes, the *beta-F1-ATPase* mRNA and its encoded protein, mitochondria and ribosomes (monosomes and polysomes) were all identified in the same fractions, supporting a co-translational import of the protein also in mammalian cells [92]. Two different elements included in the transcript (one within the coding region and the other within the 3′-UTR) were required for polysome engagement and proper protein production [92].

In neurons, we have recently identified a similar mechanism that anchors the mRNA-encoding PINK1 to the OMM. The targeting of the *Pink1* mRNA required its translation, yet the expression of only its MTS fused to BFP was not sufficient to localize the chimeric RNA to mitochondria, despite the proper import of the BFP protein [38]. Inclusion of further sequences within the ORF was necessary to also recruit the mRNA to mitochondria, arguing that these might interact with specific factors distinct from the TOM complex, such as RBPs. Indeed, we identified a complex between SYNJ2BP and a neuron-enriched splice isoform of SYNJ2 as essential factors for mitochondrial targeting of *Pink1* mRNA. Interestingly, SYNJ2 is the RBP in this case, while SYNJ2BP acts as a docking site for this recruitment, as the requirement for SYNJ2BP can be circumvented by the expression of a chimeric fusion between SYNJ2 and the SYNJ2BP transmembrane domain [38]. Also, a comparison of the transcripts binding to SYNJ2 and SYNJ2BP revealed no significant overlap [17,38], suggesting that despite the formation of a complex between the two proteins, their roles in mitochondrial mRNA targeting might be independent.

## The role of mitochondrial RNA association in neurons

Neurons are highly polarized cells, whose extreme length of up to 1 m in humans imposes neuron-specific challenges on mitochondrial homeostasis [93]. In the following chapter, we want to discuss local protein biogenesis of mitochondrial proteins and, how mitochondrial mRNA association can alleviate some of these challenges. This local availability of mRNAs may grant mitochondria far away from the soma with a certain autonomy needed for proper homeostasis and dynamic response in remote neuronal processes including both axons and dendrites. So far, most studies focus exclusively on axonal mechanisms, given their more extreme length, while generally the dendritic compartment is assumed to be less challenged. However, given the role of local translation in synaptic plasticity of the post-synaptic site and the importance of dendritic mitochondria to sustain these changes [94], we anticipate that also the dendritic association of mRNAs with mitochondria and their translation may be specifically regulated in dendrites as well.

### Local translation of mitochondrial mRNAs supports neuronal mitochondrial homeostasis

Highly polarized cells such as neurons employ subcellular transport of mRNAs to shape their local proteome [95]. Intriguingly, mitochondrial transcripts are one of the largest categories of transcripts found in axonal transcriptomes [96,97]. Indeed, local translation of individual transcripts encoding subunits of the respiratory chain and mitochondrial enzymes has been observed in neurons [96–98]. Consistently, if axonal protein synthesis is inhibited by the local application of translation inhibitors, mitochondrial activity, as measured by the membrane potential across the mitochondrial IM, is also significantly reduced [99]. Also, proteomic and transcriptomic analysis of synaptic material identified many locally translated mitochondrial proteins, which are then imported into synaptic mitochondria and integrated into the respiratory chain complexes [100]. This proportion increases upon synaptic stimulation [100], suggesting that local translation of mitochondrial proteins could serve to locally shape and alter the mitochondrial proteome to the local need. However, how are the mRNAs encoding mitochondrial proteins transported into the axon?

### Mitochondrial hitch-hiking of RNAs

**Figure 3.**
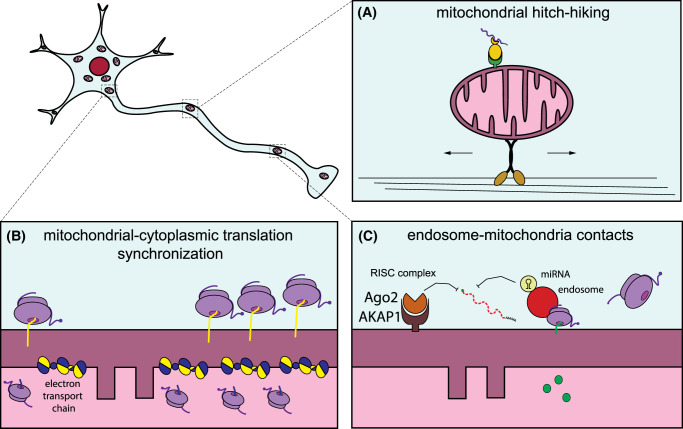
Mechanisms of mitochondrial mRNA regulation in axons. (**a**) In neurons, certain mRNA-encoding mitochondrial proteins are anchored to the mitochondrial surface by RBPs and co-migrate with them along the axons, in a process called mitochondrial hitch-hiking. Similar hitch-hiking of transcripts occurs also on endosomes and lysosomes (not shown). (**b**) For the proper assembly of the electron transport chain, the translation of mitochondrial-encoded genes from the nucleus and mitochondrial genome needs to be synchronized. (**c**) Additional regulatory processes include the compartmentalization of translation at endosomes of transcripts encoding mitochondrial proteins that will be then transferred at mitochondria–endosome contact sites. Endosomes can also transport miRNA to regulate the silencing of transcripts. Moreover, AKAP1 recruits Argonaute 2 (Ago2) to regulate mRNA decay at the mitochondrial surface.

While traditionally RNAs were thought to be transported as individual RNA granules along the neurites [101], recent studies have shown that in neurons mRNAs can also hitch-hike on various organelles, including mitochondria [38,102–104] (Figure 3a). Similar hitch-hiking on endosomes has been observed before in other polarized cells such as the fungus *Ustilago maydis* [105]. It is tempting to speculate that the mitochondrial association of mRNAs observed from yeast to flies and mammals could be used to establish cell polarity in neurons as well as in non-neuronal cells.

In neurons, mitochondria transport the mRNA-encoding the protein PINK1 via SYNJ2BP/SYNJ2 tethering, as described above [38]. The PINK1 protein is constantly degraded in healthy mitochondria, to prevent the initiation of mitophagy [106], resulting in an estimated half-life of 30 min for the PINK1 protein [107]. This short half-life is in sharp contrast with the time that it takes to reach the most distal parts of neurons: even the fastest kinds of axonal transport need days to travel the 1-m length of the axon of a motor neuron [108]. This could even be more dramatic in highly branched dopaminergic neurons, whose accumulated axonal segments would reach up to 4.5 m in humans [109]. Despite its short half-life, PINK1-dependent mitophagy is still observed in distal axons of cultured hippocampal neurons [110]. The mitochondrial association of its transcript and co-transport with mitochondria solves this conundrum and enables local PINK1 synthesis and mitophagy even in distal parts of the neuron [38]. Also, other mitochondria-associated transcripts are transported via mitochondrial hitch-hiking into the axon [38,111], despite a comparatively longer half-life of their encoded proteins. The association of mRNAs to mitochondria may, therefore, serve as a general mode of mRNA transport to ensure the availability of mitochondrial transcripts for local translation in axons. Interestingly, this mode of transport is not limited to axons, as also dendritic mitochondria carry the *Pink1* mRNA [38].

### Synchronization of mitochondrial and cytosolic translation

For the complexes of the respiratory chain, which all but complex II are a mosaic of nuclear and mitochondrial-encoded proteins, the assembly of the complexes needs to follow a concerted action of translation events of both the cytosolic and the mitochondrial ribosomes to achieve proper stoichiometry within mitochondria [112] (Figure 3b). Localized translation could be one way to synchronize complex assembly. For some examples, like the complex V subunit Atp2 in yeast, RNA association to the mitochondria is driven by its 3′-UTR and it is essential for their efficient assembly into a functional complex [91]. Analysis of ribosomal footprints of the cytosolic and the mitochondrial ribosomes upon switch from fermentable to oxidative metabolism in yeast revealed a synchronous regulation of the expression of proteins from the two genomes [113]. This is driven by the cytosolic production and import of translational activators needed for the translation within mitochondria [113]. In mammals, a similar synchrony exists between mitochondrial and cytoplasmic translation [114], yet the translational activators used to synchronize mitochondrial translation to the activity of the cytosolic ribosomes are not conserved [112]. Understanding how this gap is filled up in mammals is an intriguing field of future research. In HEK293T cells, it was shown that import of the complex IV subunit COX4 is essential to alleviate stalled translation of the mitochondrially encoded complex IV subunit COX1 [114], thereby synchronizing the mitochondrial translation to the influx of proteins from the cytosol. Assembly factors could serve a similar role, such as the recently characterized complex I assembly factor TMEM126A [115], which is also translated at the mitochondrial surface [116]. Whether the import of such factors also affects the production of mitochondrial-encoded subunits remains to be determined.

In neurons, synchronicity of mitochondrial complex assembly is especially challenging due to their high polarization and extreme length [93,117]. mtDNA is maintained all along the axon and actively replicates even in distal parts as observed by BrdU incorporation on primary neurons [117]. Metabolic labeling with non-canonical amino acids traceable by click-chemistry allowed the detection of active expression of the mitochondrial genome also in axons and dendrites [116]. Therefore, also in the distal parts of neurons, the challenging synchronization of intra- and extra-mitochondrial translation needs to be maintained. One candidate that may mediate part of the synchronization is the mitochondrial translation initiation factor 3 (mtIF3). This protein is locally translated in axons, to regulate mitochondrial translation in the neighborhood [98]. Given the confined space in the axon and the observation that most axonal translation of mitochondrial-targeted proteins occurs at mitochondria–endosome contact sites [118,119], it is likely that the *mtIF3* mRNA is located and translated close to or at mitochondria. Whether this transcript is actually bound to mitochondria, and by which means, and whether this is a prerequisite for its function in axons remains to be determined.

## Local translation supports the PINK1-mediated mitochondrial damage response

The local translation of PINK1 at the mitochondrial surface not only serves to allow PINK1/Parkin-mediated mitophagy to occur in axons, but also may itself regulate local translation of mitochondrial proteins. In *Drosophila* and HeLa cells, wild-type PINK1 kinase, but not its PD-associated kinase-dead mutant G309D, can stimulate the translation of mitochondria-associated transcripts encoding respiratory chain subunits [120]. This constitutes a mechanism by which only mildly damaged mitochondria that fail to import and degrade PINK1, can increase the local biogenesis of respiratory chain proteins to replace and repair the damage before Parkin recruitment and mitophagy occur. Mechanistically, PINK1 regulates the mitochondrial association of these transcripts, which alleviates their translational repression by the Pumilio/Pum1, a *Drosophila*/mammalian homolog of Puf3p, potentially in concert with Parkin-mediated degradation [120]. Silencing of Pum expression counteracts the effect of PINK1 G209D overexpression on mitochondrial function and morphology [120], suggesting that the PD mutant acted as a dominant negative. In line with this, it has been suggested that also in yeast, Puf3p shuttles between two complexes: a repressive complex localized in the cytosol and associated with p-bodies [121], and a translationally active complex at mitochondria [122]. The role of Pumilio proteins in mitochondrial homeostasis is, therefore, multifaceted. Intriguingly, Pum2 is necessary to prevent axonal localization of its target mRNAs [123]. How this phenotype interacts with its function as a translational repressor, and how these transcripts are transported into the axon in the absence of Pum2 remains to be determined.

In line with the function of PINK1 in mitochondrial quality control at multiple levels, PINK1 was also reported to contribute to mtDNA quality control and inheritance by altering local translation in *Drosophila* oocytes. PINK1 phosphorylates the RBP Larp, which leads to the inhibition of local protein synthesis on the mitochondrial outer membrane [124]. Therefore, only on healthy mitochondria, in the absence of PINK1 stabilization, local translation of Larp substrates can occur, promoting their import and replication of the mtDNA specifically in healthy mitochondria [124]. This mechanism reduces the transmission of deleterious mtDNA mutations to the offspring. How much this mechanism is conserved in other organisms and cell types remains to be determined. It is tempting to speculate that similar mechanisms could be in play at other cellular bottlenecks, such as the axon initial segment, to allow only healthy mitochondria to travel long distances into the axon.

### Regulation of localized translation

The abundance of a given transcript as well as its translation can be altered locally by post-transcriptional mechanisms including those mediated by the RNA-induced silencing complex (RISC) through its interaction with different sncRNAs (small noncoding RNAs). Fittingly, microRNAs (miRNAs) are detected in axonal transcriptomes [125]. Intriguingly, the Tudor domain of the mitochondrial RNA anchor AKAP1 has been reported to recruit Argonaute 2 (Ago2), an essential component of RISC [42] (Figure 3c). This recruitment brings a player of sncRNA-mediated regulation in close proximity to the RNAs localized at the mitochondrial surface, enabling their local control. It has been suggested that this mechanism may regulate the levels of the AKAP cargo *StAR*, in concert with its regulation by PKA [42], yet conclusive evidence for *StAR* mRNA cleavage or translational repression at the mitochondrial surface is still missing. Fittingly, miRNAs interacting with other mitochondrial localized transcripts have been described to localize to mitochondria. In neurons, the miRNA-338 regulates local *Cox4* levels, significantly influencing OXPHOS in axons [126]. Precursors of miRNAs hitch-hike on endosomes to reach the axonal compartment prior to their cleavage by DICER [127], so presumably they will gain access to the mitochondrial localized transcripts and the RISC complex upon formation of mitochondria–endosome contact sites. As these have been suggested to be hotspots of axonal translation of mitochondrial proteins [118,119], the regulation may coincide with the translation of the associated transcripts (Figure 3c). This is in line with the hypothesis that miRNAs can actively regulate their targets during ongoing translation (reviewed in [128]).

Also, protein phosphorylation may regulate the localization and translation of mitochondria-associated transcripts. PKA-mediated phosphorylation of AKAP1 increases its binding affinity as mentioned above, and we have recently observed that the master metabolic regulator AMP-activated protein kinase (AMPK) phosphorylates SYNJ2BP [129]. This phosphorylation is necessary for efficient recruitment of its RNA-binding partner SYNJ2 and thereby regulates the localization of *Pink1* mRNA. Intriguingly, mitochondrial localization of the transcript slows PINK1 biogenesis and subsequent mitophagy, whereas its cytoplasmically localized form is more efficient at driving mitophagy. How this is connected with the mitochondrial import of PINK1 protein remains to be determined, as well as whether the reduced availability of PINK1 is due to decreased production or increased degradation of its precursor. However, it is tempting to speculate that also co- versus post-translational import mechanisms could influence the submitochondrial localization of PINK1 and hence its proteolytic processing and mitophagy activation, analogous to fumarase import [67]. Finally, CLUH granules have been reported to recruit the kinase mammalian target of rapamycin (mTOR) in hepatocytes [84]. This interaction co-ordinates mTOR complex (mTORC) activity and mitochondrial biogenesis in response to starvation in the liver. Whether this mechanism is present in other tissues, including neurons, remains to be determined. Interestingly, the response to starvation may be dependent on the neuronal subtype or subcellular compartment, explaining controversial findings on the ability of starvation to induce autophagy in the nervous system [130]. Interestingly, Smaug granules are also sensitive to AMPK and mTOR activity, hence regulating mitochondrial activity as well [34]. In addition, the cross-talk between CLUH regulation of mTORC and AMPK-mediated regulation of PINK1 biogenesis for mitophagy will be interesting to study, given that AMPK and mTOR antagonistically regulate each other and modulate different aspects of mitochondrial biogenesis in neurons [131–133].

## The long (or short) post-translational journey to mitochondria

If the association of the mRNAs with mitochondria allows for simultaneous translation, the relative vicinity of the import channels will most likely favor co-translational import. Especially mitochondrial proteins carrying an N-terminal amphipathic MTS could engage with the receptors of the TOM complex while still being synthesized by the ribosome (see above for details). In the following chapters, we want to review the benefits and draw backs of co-translational import into mitochondria. Many of these observations have not yet been shown to be conserved in neurons, yet given the important role of mitochondrial RNA association in neurons, it is highly likely that these or similar measures will also be employed to safeguard proper mitochondrial biogenesis in neurons.

### Import problems lead to a diversity of stress responses

It is generally assumed that translation close to the mitochondrial surface will increase import fidelity and lower the chances of non-imported protein precursors accumulating in the cytosol. Especially hydrophobic membrane proteins would be highly aggregation prone and therefore toxic to the cell. Therefore, several pathways have evolved to deal with protein import failure to either degrade or reroute mitochondrial preproteins [59]. Different approaches, such as blocking the general import pore TOM40, inhibiting the intramembrane space import protein Mia40 or depleting the mitochondrial membrane potential, elicit a general stress response termed unfolded protein response activated by mistargeting of proteins (UPRam) [134–137]. This pathway is functionally conserved between yeast, *C. elegans* and mammalian cell culture systems (for an in-depth dissection and comparison see review by Tran and Van Aken [138]). The UPRam co-ordinates the reduction of cytosolic translation and biogenesis of the cytosolic ribosomal subunits, increases chaperone expression and enhances the proteasomal capacity of the cell. This serves to slow down translation and reduce the accumulation of mistargeted proteins in the cytosol. It seems likely that the absence of proper mRNA targeting to the mitochondria could elicit a similar response as well, yet this has not been experimentally shown. Finally, import stress can also contribute to induce mitophagy, which may act as a last resort to specifically remove the damaged organelle. This may occur via blocked import of the mitophagy sensor PINK1, leading to its outer membrane stalling, activation and recruitment of Parkin, as described above. In a parallel pathway, the normally matrix-targeted protein NOD-like receptor X1 (NLRX1) remains stable in the cytosol upon blockage of mitochondrial import [139]. The cytosolic location of NLRX1 enables it to bind to ribosome binding protein 1 (RRBP1) and the RBP splicing factor, proline- and glutamine-rich (SFPQ), forming a high molecular mass complex at the ER. This complex can then initiate the recruitment and lipidation of LC3 to prepare for mitophagy if the import blockage persists. While not directly associated with mitochondria, SFPQ is still involved in localizing several transcripts encoding mitochondrial proteins in neurons, by delivering them into the axon as a separate RNA granule [140]. Also, RRBP1 interacts with RNAs and ribosomes itself [141] as well as with the mitochondrial RBP SYNJ2BP [17,142]. It has been suggested that both SYNJ2BP as well as RRBP1 may preserve nuclear-encoded mitochondrial mRNAs from degradation during translational stress, analogous to cytoplasmic stress granules, in order to allow a quicker recovery of mitochondrial metabolism after stress removal [17,139]. In neurons, it may include the *Pink1* transcript, as SYNJ2BP serves as the mitochondrial anchor for the *Pink1*-binding SYNJ2 protein [38]. It is tempting to speculate that all these mechanisms may act in concert to allow local biogenesis of PINK1 and other mitochondrial proteins, regulate them in response to stress, and to elicit mitophagy by PINK1/Parkin dependent and independent mechanisms as a last resort to preserve mitochondrial function and protect the cell from non-imported mitochondrial protein precursors.

### Constant surveillance of TOM complex is required for mitochondrial homeostasis

To prevent clogging of the import system in the first place, several mechanisms survey the TOM complex and deal with stuck import intermediates or ribosomes. In yeast, the TOM import channel is associated with the ubiquitin ligase Ubx2, which ubiquitinates protein precursors clogging the TOM complex. Ubiquitinated preproteins are then removed with the help of the AAA-ATPase Cdc48 (VCP in mammals) [143] in a process called MitoTAD (mitochondrial protein translocation-associated degradation). In a parallel pathway, ubiquitinated precursor proteins can be removed by targeting of yeast ubiquillin homolog, Dsk3, to the TOM complex via its interaction with the TOM-associated peptidyl-tRNA hydrolase Pth2 [77]. In the case of the IM import, an excessive amount of IM proteins relying on the lateral release of their hydrophobic TM segments into the IM by the TIM23 complex also slow down protein import enough to elicit a stress response, which has been identified in yeast and termed mitoCPR (mitochondrial compromised protein import response; [144]). This pathway activates the transcription factor pleiotropic drug resistance 3 (Pdr3) and induces the expression of the Cis1 adaptor protein, which helps to recruit the mitochondrial AAA-ATPase Msp1 (ATAD1 in mammals) to the TOM complex. Msp1 extracts the IM protein precursors from the import channel [144]. Fittingly, proximity labeling experiments revealed that the RNAs encoding IM proteins are found particularly enriched in ribosomal footprints localized near to the mitochondrial outer membrane in yeast [13], yet how many of these RNAs carry bipartite signal sequences or how many of their encoded proteins are imported by the carrier import pathway has not been analyzed. Interestingly, a similar ribosomal proximity analysis in cultured mammalian cells detected a differential response of ribosomal association between RNAs encoding proteins carrying an MTS and RNAs encoding mitochondrial carriers upon knock down of the TOM import receptor Tom70 [14]. While the association of Tom70-dependent carrier mRNAs with mitochondrial ribosomes decreased upon Tom70 knock down as expected, MTS-containing Tom20 targets increased their association. It is tempting to speculate that this effect could be mediated by increased access to the TOM complex for MTS-containing nascent chains, arguing that cotranslational import is not only a rare phenomenon, but increasingly common under physiological conditions and that nascent chains compete for the access to the TOM complex in living cells.

Co-translational import of proteins into mitochondria, however, also presents a danger to the cell in the case that translation termination is blocked, e.g. by missing stop codons, RNA truncation or modification, or insufficient supply of certain tRNAs [145]. On cytosolic ribosomes, stalled translation is detected and mitigated by a ribosomal quality control (RQC) mechanism that induces the addition of C-terminal alanine and threonine residues as described in yeast (CAT-tails; [146]). Import of such modified mitochondrial protein precursors is toxic to the cell due to intramitochondrial protein aggregation and chaperone depletion. RQC machinery is, therefore, suppressed specifically by the interaction of Vms1 on mitochondrially localized ribosomes in yeast [147]. In *Drosophila* and cultured mammalian cells, the expression of a PD-associated PINK1 mutant impaired translation termination of mitochondrially localized transcripts. This was mediated by impairing the activity of the eukaryotic release factor 1 (eRF1) in a process called MISTERMINATE [148], consistent with the role of PINK1 as a regulator of mitochondrially associated translation [120]. PINK1 dysfunction leads to C-terminal extension (analogous to CAT-tailing) of the complex I subunit C-I30, which can be suppressed by overexpression of ANKZF1, the metazoan Vms1 homolog [148]. Mechanistically, upon uncoupling the mitochondrial membrane potential, PINK1 activity recruits the RQC components NOT4 and ABCE-1 to the mitochondrial surface [149]. NOT4 can then ubiquitinate ABCE-1, which leads to a Parkin-independent formation of ubiquitin chains on dysfunctional mitochondria [149]. Consistent with the idea that PINK1 can attract mitophagy receptors also in the absence of the feedforward amplification provided by Parkin [150], this ubiquitination serves as a mitophagy signal and leads to the removal of the damaged organelle [149]. Remarkably, overexpression of either the translation terminator eRF1 or RQC factors including NOT4 or ABCE-1 rescues PINK1 mutant phenotypes in *Drosophila* brain and muscles [148], suggesting that the translational control of C-I30 and other mitochondrially associated transcripts is one of the essential functions of PINK1. Moreover, in human samples of PD patients, the RNA levels encoding for RQC proteins including ABCE-1 are significantly down-regulated [149], suggesting that this pathomechanism may also be relevant in human neurodegenerative disease.

### An alternative route to mitochondria

Recent evidence gained from yeast has shown that mitochondrial protein precursors can also first localize to the ER membrane prior to mitochondrial import [151]. This pathway, termed ER surface retrieval (ER-SURF) serves as a backup pathway to deliver functional mitochondrial protein precursors to the organelle, presumably via mitochondria–ER contact sites (MERCS). This process depends on both the chaperone-associated protein Djp1p [151], which probably shield the hydrophobic preproteins from aggregation, as well as on the ER import pathway for the guided entry of tail-anchored proteins (GET pathway) [152], which may mediate the transfer between both organelles. The ER-SURF mechanism seems conserved in metazoans [152], although a more detailed analysis of the conservation of the individual players is still pending. A general association of ER–mitochondria to control mitochondrial protein biogenesis could also be plausible, considering the interactions of known RBPs and ribosomes with MERCS. Some examples are RRBP1 and SYNJ2BP/SFPQ (as described above), or PINK1-mediated stimulation of OMM translation, as PINK1 is also enriched at MERCS upon mitochondrial dysfunction [153]. Indeed, ER proteins are also involved in the degradation of non-imported mitochondrial protein precursors, including PINK1 [154,155]. Whether this is supported by directed targeting of mRNAs encoding mitochondrial proteins to the ER remains to be determined. Intriguingly, two recent reports suggest that the stress response of the ER, the unfolded protein response (UPR^ER^), affects mitochondrial biogenesis at the level of RNA and non-imported preproteins. Knöringer et al. [156] describe that the accumulation of un-imported mitochondrial protein precursors in yeast triggers the UPR due to their ER localization. This is in line with earlier reports that show a tight interweaving of the stress responses of the two organelles, also in mammalian cells [157,158]. Moreover, they reanalyzed datasets characterizing the proteins recognized by the ER targeting signal recognition particle (SRP) in yeast and found that a subset of mitochondrial proteins exhibit SRP binding capabilities and may, therefore, be recruited to the ER during translation. These proteins include but are not limited to proteins with dual localization signals, to both the mitochondria and the ER. Whether the activation of the UPR^ER^ is also affected by altering the localization of the transcripts encoding mitochondrial proteins is unknown, but represents an intriguing possibility for the cell to favor mitochondrial import over ER targeting. While yeast cells only message ER stress via activation of Ire1, mammals have evolved two further arms of the UPR^ER^ to regulate the transcription of proteostasis genes and slow down translation [159]. One of these arms uses the ER-localized kinase PERK to phosphorylate eIF2a, which then attenuates cytoplasmic translation [158]. Hughes et al. [160] now report that mitochondrial localized transcripts are exempt from the global translational repression due to a direct interaction between PERK and the OMM protein ATAD3a. How this affects the targeting of the encoded proteins has not been assessed. While on the one hand, it seems obvious that selective ER stress might benefit from continued mitochondrial biogenesis to ensure ATP supply to support the action of chaperones, the entanglement of mitochondrial and ER protein biogenesis suggests that this view may be too simplistic. However, definitive evidence for the direct conservation of the ER-SURF pathway in mammals and more specifically in neurons is still pending.

## Outlook

Mitochondrial RNA association is a conserved feature from yeast to higher eukaryotes. In complex cells such as neurons, this serves to support the local translation of proteins necessary for mitochondrial function, including the biogenesis and assembly of the respiratory chain and the repair or elimination of damaged mitochondria. Consistently, dysregulation of local translation leads to mitochondrial damage and is associated with neuronal phenotypes in cultured neurons, flies and mice [99,149,161]. Future research will need to address how this in turn affects neuronal biology and function. Mitochondria are emerging as platforms for local translation and its regulation in neurons thereby can influence neuronal morphology and physiology [94,162]. Understanding the various ways how RNAs can tether to mitochondria, as well as insights into the regulation of their local translation will allow us to make the leap from biochemical associations to functionality both for neuronal and organismal health. Finally, upcoming new methods such as the targeted isolation of mitochondria from specific neuronal subtypes [94] or the targeted identification of RNAs using light-activated proximity biotinylation approaches [163] will allow us to gain a more detailed view of which transcripts are associated with mitochondria at any given time or in any given neuron. Both options, the localization of all transcripts needed to locally double mitochondrial mass, or the select enrichment of only a given subset, would greatly change how we view mitochondrial biogenesis in neurons and provide new therapeutic targets to counteract mitochondrial dysfunction in aging and disease.

## Open Access

Open access for this article was enabled by the participation of Max Planck Digital Library in an all-inclusive *Read & Publish* agreement with Portland Press and the Biochemical Society under a transformative agreement with MPDL.

## Abbreviations

AMPK: AMP-activated protein kinase
ER: endoplasmic reticulum
eRF1: eukaryotic release factor 1
FMRP: Fragile X mental retardation protein
GPX4: glutathione peroxidase 4
IM: inner membrane
LARP4: La-related protein 4
LPL: lipoprotein lipase
MCP: MS2-coat protein
MERCS: mitochondria–ER contact sites
MFF: Mitochondria fission factor
miRNA: microRNA
mtDNA: mitochondrial DNA
mtIF3: mitochondrial translation initiation factor 3
mTOR: mammalian target of rapamycin
mTORC: mTOR complex
MTS: mitochondrial targeting signal
NAC: nascent polypeptide-associated complex
NLRX1: NOD-like receptor X1
OMM: mitochondrial outer membrane
OM14: outer membrane protein 14 kDa
OXPHOS: oxidative phosphorylation
PD: Parkinson's disease
PINK1: PTEN-induced kinase 1
PKA: protein kinase A
RBP: RNA-binding protein
RISC: RNA-induced silencing complex
RNP: ribonucleoprotein
RPL3: ribosomal protein of the large subunit 3
RQC: ribosomal quality control
RRBP1: ribosome binding *protein* 1
SAM37: sorting and assembly machinery 37
SFPQ: splicing factor, proline- and glutamine-rich
SRP: signal recognition particle
StAR: steroidogenic acute regulatory
TCA: tricarboxylic acid cycle
TIM: translocase of the inner membrane
TOM: translocase of the outer membrane
